# Modifying the Non-muscle Invasive Bladder Cancer Immune Microenvironment for Optimal Therapeutic Response

**DOI:** 10.3389/fonc.2020.00175

**Published:** 2020-02-18

**Authors:** Nicola E. Annels, Guy R. Simpson, Hardev Pandha

**Affiliations:** Department of Clinical and Experimental Medicine, Faculty of Health and Medical Sciences, University of Surrey, Guildford, United Kingdom

**Keywords:** bladder cancer, microenvironment, immunotherapy, immunomodulation, immunity

## Abstract

It is now well-recognized that the tumor microenvironment (TME) is not only a key regulator of cancer progression but also plays a crucial role in cancer treatment responses. Recently, several high-profile publications have demonstrated the importance of particular immune parameters and cell types that dictate responsiveness to immunotherapies. With this increased understanding of TME-mediated therapy, approaches that increase therapeutic efficacy by remodeling the TME are actively being pursued. A classic example of this, in practice by urologists for over 40 years, is the manipulation of the bladder microenvironment for the treatment of non-muscle invasive bladder cancer (NMIBC) by instillation of intravesical bacillus Calmette-Guerin (BCG). The success of BCG treatment is thought to be due to its ability to induce a massive influx of Th1-polarized inflammatory cells, production of Th1 inflammatory cytokines and the generation of tumor-targeted Th1-mediated cytotoxic responses. Whilst BCG immunotherapy is currently the best treatment for NMIBC, ~30% of patients show no response to this treatment. Here we present a review highlighting a variety of promising alternative immunotherapies being developed that remodel the bladder tumor microenvironment. These include (1) the use of oncolytic viruses which selectively replicate within cancer cells whilst also modifying the immunological components of the TME, (2) manipulation of the bladder microbiome to augment the response to BCG or other immunotherapies (3) utilizing Toll-like Receptor agonists as anti-tumor agents due to their potent stimulation of innate and adaptive immunity and (4) the growing recognition that immunotherapeutic strategies that will have the largest impact on patients may require multiple therapeutic approaches combined together. The accumulating knowledge on TME remodeling holds promise for providing an alternative therapy for patients with BCG-unresponsive NMIBC.

## Introduction

In addition to malignant cells the TME is also made up of other non-transformed cells and secreted extracellular components. The interactions between the tumor cells and the tissue microenvironment are such that they regulate tumor progression but also determine cancer treatment responses. In particular, the tumor microenvironment often limits the infiltration and function of effector T cells into the tumor, recruiting myeloid derived suppressor cells (MDSCs), tumor associated macrophages (TAMs) and regulatory T cells (Tregs), providing an immunosuppressive niche to help cancer cells escape from immune surveillance (1, 2). Understanding the complex interplay of the components that make up the tumor microenvironment can help inform on strategies to modulate the tumor microenvironment to be more immunogenic resulting in enhanced immune responses and improved therapeutic outcomes.

## The Gold Standard Immunomodulatory Approach to Treat NMIBC: Intravesical BCG

One of the oldest such immunomodulatory approaches and the gold standard treatment for non-muscle invasive bladder cancer is serial intravesical instillations with bacillus Calmette-Guerin (BCG) (3, 4). Effective BCG therapy has been shown to prevent or delay tumor recurrence and progression and this has been attributed to its ability to induce a massive influx of inflammatory cells (Th1-polarized lymphocytes and neutrophils), the generation of classically activated resident tissue macrophages (M1), the production of Th1 cytokines (IFNγ, IL-12, and TNF-α) and the generation of anti-tumor targeted Th1-mediated cytotoxic responses (5–7). However, around 30% of patients are unresponsive to BCG therapy and increasing evidence points toward the pre-existing immune tumor microenvironment influencing the BCG response (8).

As is the case for many solid tumors, the tumor microenvironment of non-muscle invasive bladder cancer is characterized by the presence of pro-inflammatory cells (such as macrophages, myeloid-derived suppressor cells, regulatory T cells, dendritic cells, mast cells, neutrophils, and lymphocytes) and cytokines (such as tumor necrosis factor-α and interleukins) both in the supporting stroma and in tumor areas. Many of these cell types have been studied to investigate their influence on outcomes following BCG immunotherapy. Several published reports have provided evidence that a predominance of immunosuppressive cell types are associated with BCG immunotherapy failure. In particular, the presence of M2-like tumor associated macrophages (TAMs) has consistently been associated with shorter recurrence-free survival and thus a poor response to BCG (7, 9–11). In NMIBC these TAMs are induced and maintained by Bone Morphogenetic Protein 4 (BMP4) secreted by the tumor cells (12). They are able to suppress adaptive immunity, support tumor growth and angiogenesis and aid cell migration, invasion and metastasis (13).

Whilst the TAMs are localized in the stroma-tumor margin of NMIBC, infiltrating the tumor area in high-grade tumors, another immunosuppressive cell type the Tregs localize in the stroma around the cancer lesion regardless of tumor stage and grade (9). High Treg counts were shown to be an independent predictor for recurrence following BCG treatment (7, 9). Whilst the above studies focused on immunohistochemical analysis of the NMIBC tissue microenvironment, another study characterized immune cell populations in the urine of patients undergoing BCG instillations for the treatment of NMIBC as a surrogate for the bladder tumor microenvironment (14). They observed an infiltration of neutrophils, T cells, monocytic myeloid-derived suppressor cells (M-MDSCs) and group 2 innate lymphoid cells (ILC2), cells that have been shown to play a part in regulating tissue homeostasis during infection, chronic inflammation, metabolic disease and cancer (15). There was a lower recurrence-free survival in patients with a T cell-to-MDSC ratio of <1 than in patients in which the ratio was >1. This difference between patient groups was even present before BCG therapy. Bladder tumor cells cultured *in vitro* with BCG could shift ILCs toward the ILC2 phenotype producing the Th-2 cytokine IL-13 which allowed the recruitment and immunosuppressive function of monocytic cells.

Amongst the robust immune response induced by BCG therapy, *in vitro* and *in vivo* studies have suggested a role for NK cells in BCG-induced cytotoxicity (16–18). Brandau et al. showed *in vitro* that BCG-activated killer (BAK) cells, of which NK cells were the major effector cell population, displayed substantial cytotoxicity against bladder tumor cells. Furthermore, using a syngeneic orthotopic murine bladder cancer model they demonstrated in NK-deficient beige mice and in mice treated with anti-NK1.1 monoclonal antibody that BCG therapy was completely ineffective, suggesting a key role for NK cells during BCG immunotherapy (19).

As well as detecting a diverse infiltrate of innate and adaptive immune cells including the above mentioned cell types, another study reported a significant role for IL-17 positive mast cells in influencing the outcomes from BCG therapy (20). Patients with carcinoma *in situ* (CIS, high grade cancer cells that are only in the innermost layer of the bladder lining) with higher numbers of IL-17+ mast cells showed significantly longer event-free survival after intravesical BCG therapy than patients with less IL-17+ mast cells. This significant effect was only observed in patients who underwent intravesical BCG treatment suggesting that BCG amplifies the beneficial effects associated with increased numbers of IL-17+ mast cells.

Clearly the interactions between cancer and immunity are highly complex and multifactorial and in particular we still need to have a better understanding of the mechanisms hindering efficient constitutive and/or treatment-induced immune responses to tumors. Currently, in NMIBC, there are no reliable biomarkers which allow prediction of the efficacy of the BCG induced anti-tumor response despite many attempts to look at the immune response in bladder tissue before and after BCG treatment. This may be reflective of the fact that many of the studies to date looking at the immune response to BCG treatment have focused on individual cell types using traditional single immunohistochemical stains rather than performing a multiplex comprehensive analysis of both cellular and non-cellular immune components. With the introduction of new technologies that allow for a more global analysis of complex disease states a more comprehensive picture of the roles and interactions between these different components and their influence on therapies should be revealed. This will allow for more informed treatment strategies on how best to immunomodulate particular bladder cancer microenvironments to achieve the optimal therapeutic outcomes.

## The Urinary Microbiome: A Potential Emerging Factor in the Immunomodulation of NMIBC

Whilst urologists have been using BCG for over 40 years to manipulate the bladder microbiome to treat NMIBC, the patient's own pre-existing bladder microbiome may have a role not only in the development of bladder cancer but also in its response to immunotherapies (21). A greater understanding of the potential dysbiosis–the imbalance or alteration of bacterial composition of microbiota–in bladder cancer could lead to the bladder microbiome of patients being used as a modifiable way to optimize response to immunotherapy. Research to date suggests that the bladder microbiome may modulate the bladder microenvironment by various mechanisms. Firstly, bacterial strains have been shown to reduce mucosal inflammation due to inhibition of the NF-κB pathway, IL-6, and IL-8 (22). This action could affect immunotherapies e.g., BCG that rely on the initiation of a local inflammatory response. Furthermore, other studies have shown that certain bacterial species such as *Lactobacillus iners*, one of the prevalent genera detected in the human urinary microbiome (23), may be superior at binding fibronectin thus out-competing BCG whose activity relies on binding to urothelial fibronectin (24). To date, there have been few studies which have looked at the role of the urinary microbiome in bladder cancer. One of the first studies comparing the microbiome of urine specimens from healthy individuals (*n* = 6) versus urothelial carcinoma patients (*n* = 8) using 16S sequencing found that the abundance of the genus Streptococcus was most often significantly elevated in urothelial carcinoma patients (25). In contrast, Popovic et al. reported no significant differences in microbial diversity or overall microbiome composition in a study comparing the voided urine of 12 UCC patients to 11 controls using 16S sequencing, although did note that *Fusobacterium*, a genus associated with colorectal cancer, was enriched in the bladder cancer group (26). A third study, comparing 31 male UCC patients to 18 healthy controls using 16S sequencing of midstream voided urine showed an enrichment of some bacterial genera (*Acinetobacter, Anaerococcus*, and *Sphingobacterium*) and decrease of other bacterial genera (*Serratia, Proteus*, and *Roseomonas*) in the cancer group when compared to the non-cancer group. A further finding was the enrichment of *Herbaspirillum, Porphyrobacter*, and *Bacteroides* observed in cancer patients with high risk of recurrence and progression indicating that these genera may be potential biomarkers for risk stratification (27). Given the data on gut microbiota in modulating sensitivity to immune checkpoint inhibitors in advanced cancer patients (28–31) further studies investigating the influence of urinary microbiota on the bladder tumor response to anti-cancer therapy should be pursued. The studies performed to date whilst providing some preliminary interesting findings are limited due to the low numbers of samples studied and their use of voided urine which introduces contamination from microorganisms in the terminal portion of the urethra. Clearly larger scale future studies using catheterized urine need to be conducted to accurately evaluate the potentially important role of the bladder microbiome in both bladder cancer pathogenesis/progression and response to immunotherapy agents.

## Oncolytic Virus Therapy: Converting “Immunologically Cold” Tumors into Inflamed “Hot” Tumors

One intervention capable of dramatically altering the TME immune landscape, is the use of oncolytic or “cancer-killing” viruses (OVs) (32). OVs lead to improved anti-tumor immunity through the induction of both innate and adaptive immune responses, releasing the full range of tumor-associated antigens (TAAs) into an inflammatory environment via tumor lysis and the induction of immunogenic cell death, disrupting the immunosuppressive TME (33–36). Therefore, OVs are able to vaccinate against the entire range of TAAs and together with epitope spreading in the TME act as a personalized immunotherapeutic. Considerable evidence has shown that OVs are a very promising strategy to convert non-inflamed or “cold” tumors into an inflamed or “hot” phenotype to promote the priming of anti-tumor immune responses. To date, two studies have clearly shown the promise of this immunotherapeutic strategy to treat NMIBC. Firstly, preclinical work from our own group had indicated the sensitivity of bladder cancer cell lines to a novel oncolytic virus, Coxsackievirus A21 (CVA21) (37). CVA21 is able to target, infect and lyse cells expressing the CVA21 cellular receptors intercellular adhesion molecule-1 (ICAM-1) and decay-accelerating factor (DAF) (38). Infection of bladder cancer cell lines by CVA21 led to the induction of immunogenic cell death in CVA21-treated cell lines giving promise to the potential clinical translation of these results to generate long-lasting protective anti-tumor immunity in the bladder mucosa (37). These results provided the rationale for a Phase I/II clinical trial (CANON) to investigate the therapeutic potential of CVA21 as a new immunotherapy approach for the treatment of NMIBC (39). This trial determined safety, feasibility and immunomodulatory effects of CAVATAK in treatment naive tissue following escalating intravesical doses of a novel bio-selected formulation of CVA21 (CAVATAK) administered alone or in combination with mitomycin C (previously shown to up-regulate the viral entry receptor ICAM-1) (37) in 15 first-line NMIBC patients prior to TURBT surgery. Clinical activity of CAVATAK was highly tumor-selective and demonstrated the ability to induce tumor inflammation and hemorrhage following either single or multiple administrations of CAVATAK in several patients, and led to a complete resolution of tumor in one patient. Whether used alone or in combination with mitomycin C, CAVATAK caused marked inflammatory changes within NMIBC tissue biopsies by upregulating interferon-inducible genes including both Th1-associated chemokines and immune checkpoint inhibitory genes (PD-L1 and LAG3) supporting future combination studies with immune checkpoint inhibitors (40–42).

A second notable study in this field was the use of CG0070, a conditionally replicating oncolytic serotype 5 adenovirus (Ad5) designed to preferentially replicate in and kill retinoblastoma (Rb) pathway defective cells using the E2F-1 promotor (43). To enhance longlasting antitumor immunity, CG0070 encoded the cDNA for the human cytokine, granulocyte macrophage-colony stimulating factor (GM-CSF) which was selectively produced in Rb pathway–defective tumor cells due to the dependence of the E3 promoter that drives GM-CSF expression on transactivation by E1A. Preclinical *in vivo* studies with CG0070 demonstrated strong anti-tumor activity of the virus in bladder transitional cell carcinoma xenograft tumor models and showed significant anti-tumor synergy when combined with the chemotherapeutic agent docetaxel (43). In a phase 1 trial of 35 patients who had previously failed BCG therapy, CG0070 was administered intravesically in single or multiple doses at various levels (44). High levels of GM-CSF were identified in the urine of all patients after administration and no adverse events related to treatment were reported. The overall response rate to CG0070 was 48.6% (17 of 35), which improved to 63.6% (14 of 22) in the multi-dose group. Interim results from an ongoing phase 2 multicenter trial (NCT02365818) of intravesical instillation of CG0070 in 45 NMIBC patients who failed BCG therapy and had refused radical cystectomy showed an overall complete response of 47% (21/45) (45). Unfortunately, neither of the CG0070 clinical trials reported any immunohistochemical analysis of the tumor microenvironment to fully elucidate both the mechanism of action of CG0070 and potential immune activation. However, it is presumed that CG0070 works through direct tumor lysis by selective replication in Rb pathway-defective tumor cells and through immune-mediated killing resulting from immunogenic cell death and immune activation induced by the local GM-CSF production (45). Importantly, intravesical oncolytic virus therapy was extremely well-tolerated in all of the above clinical trials and thus may offer an alternative to the 40-year-old standard of care, BCG therapy, but without its limiting toxicities.

## Combination Approaches: Personalized Immunomodulation of a Patient's Tumor for Enhanced Therapeutic Outcome

Whilst the emergence of new immuno-oncology therapies has improved the survival rates of patients, particularly in hard to treat cancers, most cancer patients still either don't respond fully to immunotherapy agents or become resistant. This is in large part due to a lack of understanding of the tumor microenvironment of each patient's tumor to enable the correct immunotherapy approach to be targeted to the right tumor at the right time. It is becoming increasingly recognized that this will require the use of rational combination approaches so that more patients will respond and for longer. There are now a variety of rational combinations of immunotherapy and targeted agents which are also now being investigated in NMIBC. One such combination approach is the use of immune checkpoint inhibitors in combination with BCG. This is currently being evaluated in both a phase I study (NCT02324582) of pembrolizumab (antibody which targets and blocks PD-1) in combination with BCG for patients with high-risk NMIBC (46) and Atezolizumab (antibody against PD-L1) with or without BCG (NCT02792192). Inman et al. had already provided the evidence to support the rationale for such combination by demonstrating that PD-L1 expression is associated with high-grade tumors and intratumoral lymphocytic infiltration and was a key determinant of stage progression (47). In addition, PD-L1 expression was shown to be abundant in BCG induced bladder granulomata in 11/12 patients who failed BCG treatment, highly suggestive of a role for tumor PD-L1 in attenuating responses to BCG immunotherapy by inhibiting any anti-tumor T cells. More recently further preclinical work confirmed that PD-L1 expression was obviously upregulated in bladder cancer cells in response to BCG treatment both *in vitro* and *in vivo* (48). Wang et al. reported that treatment with a combination of BCG and anti-PD-L1 resulted in an enhanced anti-tumor effect in an orthotopic rat bladder cancer model by reducing tumor burden and prolonging survival. The antitumor immunity was attributed to an increase in the number and activity of tumor-infiltrating CD8+ T cells, as well as suppression of MDSCs in the TME (48).

Another promising immunotherapy agent currently being trialed in different immunotherapy combination strategies is an IL-15 superagonist, ALT-803, that has a proven potent ability to expand and functionally activate both NK cells and T-cells (49). This IL-15 superagonist has already shown significant anti-tumor activity as a monotherapy against various solid tumor models (50, 51), however, used in combination with e.g., BCG has the potential to further augment the BCG-induced immune response. Indeed, in a bladder cancer rat model a 46% reduction in tumor burden in response to intravesical ALT-803 and BCG combination therapy (compared to 15% with BCG alone and 35% with ALT-803 alone) was linked to increased production and secretion of IL-1α, IL-1β, and RANTES, which in turn, induced the proliferation and activation of NK cells. This enhanced therapeutic index seen with BCG and ALT-803, administered subcutaneously or intravesically (52, 53), provided a powerful justification for the ongoing current Phase Ib/II, multicenter study of intravesical BCG plus ALT-803 in high risk NMIBC (NCT02138734) (54).

The use of low-dose chemotherapy that elicits immune-potentiating effects by either inducing immunogenicity or relieving tumor-induced immunosuppression is another strategy being pursued to immunomodulate the tumor microenvironment. Certain chemotherapeutic agents not only have direct anti-tumor effects but have also been shown to play a role in depleting regulatory T cells (55), upregulating major histocompatibility complex class I expression and thus directly stimulating T cell function (56) as well as myeloid-derived suppressor cell (MDSC) depletion (57), increasing the level of type I interferons (58) and induction of immunogenic cell death (59). This “chemo-immunotherapy” strategy was utilized to immunologically evaluate the efficacy of intravesical chemotherapeutic agents, mitomycin C (MMC) or Adriamycin (ADM) combined with BCG using an N-butyl-N-(4-hydroxybutyl) nitrosamine (BBN)-induced orthotopic bladder cancer model. Hori et al. showed that sequential treatment with BCG and chemotherapy inside the bladder was more effective than either agent alone (60). The synergy was mediated through direct cytotoxic effects and indirectly through changes to immune cells through recruitment of NK cells and inhibition of TAMS and Tregs in the TME. Therefore, intravesical chemotherapy was able to suppress protumoral immunity and enhance anti-tumoral immunity in turn increasing the efficiency of BCG and potentially being a novel treatment strategy for BCG-failure NMIBC.

To date the use of vaccine therapy as a monotherapy in BCG-refractory NMIBC patients has shown disappointing clinical outcomes suggesting that this approach could be optimized by combining vaccination with local immunostimulation. One such approach to enhance a vaccine-induced immune response is through the use of Toll-like receptor (TLR) agonists that are able to modify the expression of selectins, integrins, chemokines, and chemokine receptors, thus enhancing T-cell attraction to the tumor site (61, 62). Domingos-Pereira et al. used an orthotopic model expressing E7 as a prototype tumor antigen and a cognate E7 vaccine to explore the ability of either synthetic or bacterial intravesical instillation of synthetic toll-like receptor (TLR) agonists to increase CD8 T-cell recruitment to the bladder and improve bladder tumor regression (63). They showed that immunostimulation with Ty21a bacteria (attenuated *Salmonella enterica* typhi Ty21a live vaccine-strain against typhoid fever), but not CpG, after tumor antigen vaccination efficiently recruits vaccine-specific CD8 T cells to the bladder, resulting in tumor regression and 90% survival of the mice. Salmonella can provide TLR-4 (64) and TLR-9 agonists (65) and may engage TLR-5 through flagellin (66). In a more recent preclinical study the same authors demonstrated that whilst intravesical Ty21a induced tumor cell death and innate and adaptive immune responses in the same therapeutic line as BCG immunotherapy, Ty21a was more effective than BCG for bladder-tumor treatment and thus may be predictive of a higher efficacy in patients (67).

In a phase 1 clinical trial the activity of TMX-101, a liquid formulation of the toll-like receptor 7 agonist, imiquimod, was analyzed in low-grade NMIBC (68). Whilst the effective biologic dose in this phase 1 study could not be determined because no patient experienced a complete response, the safety of TMX-101 was confirmed. A phase 2 study using TMX-101 in patients with NMIBC containing carcinoma-*in-situ* reported results from 12 patients, of which half (6/12) had received ≥2 prior induction courses of BCG (69). TMX-101 was found to be safe and well-tolerated. Two patients demonstrated a negative cytology and biopsy result at 6 weeks following treatment. Following treatment there was a significant increase in urinary cytokines, including IL-6 and IL-18. It is clear given these encouraging results that further investigations with this agent are required. However, despite the immunostimulatory potential of TLR agonists their use in cancer has been decreasing (70). Perhaps in the future these agents can be combined with other immunotherapeutic treatments in a safe and efficient way in order to achieve enhanced anti-tumor responses in patients.

## Conclusions

The tumor microenvironment has a profound impact on the success or failure of treatments for NMIBC. There is a growing realization that remodeling the tumor microenvironment to achieve optimal therapeutic effects will require multiple complementary therapeutic approaches. At the core to achieving this is a critical understanding of the bladder tumor microenvironment including the influence of the bladder microbiome in NMIBC both during tumor progression and in response to treatment. Only with this knowledge can we optimally make use of novel emerging immunotherapies and how they can complement existing therapies to achieve alternative bladder-sparing options critically needed in patients with BCG unresponsive NMIBC.

## Author Contributions

NA wrote the main body of the text. GS contributed to the oncolytic virus section. HP reviewed and edited the manuscript.

### Conflict of Interest

The authors declare that the research was conducted in the absence of any commercial or financial relationships that could be construed as a potential conflict of interest.
